# Supplementary material for: Exploring drivers of unsafe disposal of child stool in India using hierarchical regression model
Source: PLoS One. 2024 Mar 18;19(3):e0295788. doi: 10.1371/journal.pone.0295788 (PMC10947681; doi:10.1371/journal.pone.0295788)
Supplement: S1 Table — (DOCX) [file pone.0295788.s001.docx]

S1 Table. Operational description of the predictor variables

| **Independent variables** | **Operational description** | **Coding** |
| --- | --- | --- |
| Mother’s age | The age of the respondents is divided into 4 categories | 15-19 years (1), 20-24 years (2), 25-29 years (3), ≥30 years (4) |
| Mother’s education | Mother’s education is classified into four categories depending on years of schooling: No education = no years of schooling; primary = 1–5 years of schooling; secondary = 6–10 years of schooling; and above secondary = more than 10 years of schooling. | No education (0), Primary (1), Secondary (2), Higher (3) |
| Religion | Religious follower is classified into four categories | Hindu (1), Muslim (2), Christian (3), Others (4) |
| Social group | Social group/Caste is classified into five classes | General (1), SC (2), ST (3), OBC (4), Don’t know (5) |
| Wealth quintile | Economic status of the respondents' households and divided into five categories | Poorest (0), Poor (1), Middle (2), Rich (3), Richest (4) |
| Mass media exposure | Exposure to mass media was assessed from the frequency of reading newspapers/magazines, watching television, and listing radio. On the basis of these three media, respondents were categorized into three groups: low exposure (none of these media accessed), partial exposure (access to one or two media), and high exposure (access to all three mass media) | Low exposure (1), Partial exposure (2), High exposure (3) |
| Water facility at premises | Water facility is classified into two categories | Yes (1), No (2) |
| Sanitation facility | Sanitation facility is classified into three categories as per demographic health survey (DHS) guideline | Improved (1), Unimproved (2), No facility/open defecation (3) |
| Place of residence | Place of residence is classified into two categories | Urban (1), Rural (2) |
| Geographical region | India has been divided into 6 regions based on geographical and cultural settings. These are I. North region (Jammu and Kashmir, Himachal Pradesh, Punjab, Haryana, Chandigarh, Rajasthan, Delhi), II. Central region (Uttar Pradesh, Madhya Pradesh Chhattisgarh and Uttarakhand), III. East region (Bihar, Jharkhand, West Bengal and Orissa), IV. Northeast region (Arunachal Pradesh, Assam, Manipur, Meghalaya, Mizoram, Nagaland, Sikkim, and Tripura), V. West region (Gujarat, Maharashtra, Goa, Dadra Nagar Haveli and Daman Diu), VI. South region (Andhra Pradesh, Karnataka, Kerala, Tamil Nadu Puducherry, Telangana, Andaman Nicobar, Lakshadweep). | North (1), Central (2), East (3), Northeast (4) West (5), South (6), |
